# Effects of lactic acid bacteria inoculants on the nutrient composition, fermentation quality, and microbial diversity of whole-plant soybean-corn mixed silage

**DOI:** 10.3389/fmicb.2024.1347293

**Published:** 2024-04-15

**Authors:** Junzhao Xu, Jianfei Ma, Rula Sa, Humujile Sui, Xiaoni Wang, Qi Li, Xinyue Zhu, Baiyila Wu, Zongfu Hu, Huaxin Niu

**Affiliations:** College of Animal Science and Technology, Inner Mongolia Minzu University, Tongliao, China

**Keywords:** silage, *Lactobacillus plantarum*, *Lactobacillus buchneri*, aerobic stability, microbial community

## Abstract

The mixture of whole-plant soybean and whole-plant corn silage (WPSCS) is nutrient balanced and is also a promising roughage for ruminants. However, few studies have investigated the changes in bacterial community succession in WPSCS inoculated with homofermentative and heterofermentative lactic acid bacteria (LAB) and whether WPSCS inoculated with LAB can improve fermentation quality by reducing nutrient losses. This study investigated the effect of *Lactobacillus plantarum* (*L. plantarum*) or *Lactobacillus buchneri* (*L. buchneri*) on the fermentation quality, aerobic stability, and bacterial community of WPSCS. A 40:60 ratio of whole-plant soybean corn was inoculated without (CK) or with *L. plantarum* (LP), *L. buchneri* (LB), and a mixture of LP and LB (LPB), and fermented for 14, 28, and 56 days, followed by 7 days of aerobic exposure. The 56-day silage results indicated that the dry matter content of the LP and LB groups reached 37.36 and 36.67%, respectively, which was much greater than that of the CK group (36.05%). The pH values of the LP, LB, and LPB groups were significantly lower than those of the CK group (*p* < 0.05). The ammoniacal nitrogen content of LB was significantly lower than that of the other three groups (*p* < 0.05), and the ammoniacal nitrogen content of LP and LPB was significantly lower than that of CK (*p* < 0.05). The acetic acid content and aerobic stability of the LB group were significantly greater than those of the CK, LP, and LPB groups (*p* < 0.05). High-throughput sequencing revealed a dominant bacteria shift from Proteobacteria in fresh forage to Firmicutes in silage at the phylum level. *Lactobacillus* remained the dominant genus in all silage. Linear discriminant analysis effect size (LEFSe) analysis identified *Lactobacillus* as relatively abundant in LP-treated silage and *Weissella* in LB-treated groups. The results of KEGG pathway analysis of the 16S rRNA gene of the silage microbial flora showed that the abundance of genes related to amino acid metabolism in the LP, LB, and LPB groups was lower than that in the CK group (*p* < 0.05). In conclusion, LAB application can improve the fermentation quality and nutritional value of WPSCS by regulating the succession of microbial communities and metabolic pathways during ensiling. Concurrently, the LB inoculant showed the potential to improve the aerobic stability of WPSCS.

## 1 Introduction

The increasing demand for pasture-based feeds with acceptable protein content is crucial due to China’s growing demand for meat and milk, exacerbating the feed shortage in the ruminant industry (Meng et al., 2022). Whole-plant soybeans, as legumes, offer high yields, ease of harvest, and high protein and vitamin content with good palatability and digestibility (Gandra et al., 2022). However, their lower water-soluble carbohydrate (WSC) content and higher buffering energy lead to higher pH in whole-plant soybean silage, limiting LAB growth and challenging good silage production (Ghizzi et al., 2022). In contrast, whole-plant corn, a graminaceous plant, serves as a primary roughage source in many countries. Its low buffering energy and high WSC content facilitate high-energy silage production. Nevertheless, its low crude protein (CP) content necessitates additional protein sources, like soybean meal or cake, for ruminant feed (Meng et al., 2022). Prior research has demonstrated superior overall silage quality in the corn-soybean strip-intercrop model compared to corn or soybean silage alone (Ni et al., 2018; Zeng et al., 2020; Meng et al., 2022). Kizilsimsek et al. (2017) reported that silage with a whole soybean to whole corn ratio of 40:60 exhibited better fermentation quality and superior aerobic stability than soybean silage alone compared to corn silage. The above studies confirmed the viability of mixed whole-plant corn and soybean silages. However, they also highlight problems such as high levels of ammonia nitrogen (NH_3_-N) resulting from protein degradation and elevated yeast counts in silage, which compromise aerobic stability, and pH levels above 4.5, among other concerns (Kizilsimsek et al., 2017; Zeng et al., 2020). Therefore, this study investigated the use of lactic acid bacteria (LAB) inoculants to reduce nutrient loss and improve fermentation quality in the WPSCS process.

*Lactobacillus plantarum* is a homofermentative LAB favored in silage production, metabolizing WSC in silage to lactic acid and rapidly lowering pH to inhibit yeast and mold growth (Mu et al., 2020). Inoculation with *L. plantarum* in mixed alfalfa and Leymus chinensis silage improves fermentation quality by increasing LA content and reducing pH and NH_3_-N content (Si et al., 2023). In contrast, *L. buchneri*, a heterofermentative LAB, enhances silage’s aerobic stability by converting LA into acetic acid (AA), thereby inhibiting filamentous fungi growth (Yin et al., 2023). da Costa et al. (2022) reported that *L. buchneri* inoculation increased AA concentration and reduced yeast and ethanol content in sugarcane and peanut silages. Current research on whole-plant corn-soybean predominantly focuses on the intercropping model and the corn-soybean ratio during silage processing (Kizilsimsek et al., 2017; Ni et al., 2018; Zeng et al., 2020; Meng et al., 2022), and the above two mixed silage methods are also limited by nutrient loss and incomplete fermentation. To date, few studies have investigated the effects of homofermentative and heterofermentative lactic acid bacteria inoculation on changes in the quality, microbial composition, and expected functions of WPSCS. We hypothesize that LAB inoculation will induce changes in the succession of bacterial communities within mixed silages and improve the fermentation quality of WPSCS by reducing ammonia nitrogen levels and pH in mixed silages. This study evaluated the effect of adding *L. plantarum* or *L. buchneri* inoculant on the fermentation quality and nutrient composition of whole-plant soybean–corn mixed silage and analyzed the bacterial community and functional prediction to gain a deeper understanding of LAB.

## 2 Materials and methods

### 2.1 Materials and silage preparation

On September 14, 2022, fresh whole soybeans (R5 stage, seed fullness 1/2 ~ 3/4) and whole corn (wax ripe stage) were manually harvested from the soybean-corn strip intercropping field of the Institute of Agriculture and Animal Husbandry Science, Tongliao, Inner Mongolia Autonomous Region, China. The soybean stubble was maintained at a height of 5 cm, and the corn stubble at 15 cm. The harvested plants were transported to the laboratory and chopped into 1–2 cm lengths using a crop cutter. Chemical characterization and microbial counts were determined after thoroughly mixing stems and leaves (Table 1). *Lactobacillus plantarum* (No. 6026) and *Lactobacillus buchneri* (No. 20294), isolated from fermented total mixed rations, were used as inoculants. The fresh material (FM) was mixed in a 40:60 ratio by fresh weight. *L. plantarum* (LP), *L. buchneri* (LB), and the combined inoculant (LPB, 1:1) were applied at a rate of 1 × 10^−6^ cfu/g FM, dissolved in 20 mL of distilled water, and sprayed evenly on the FM. The control group (CK) received 20 mL of distilled water without inoculants. A total of 500 g FM was placed in vacuum bags (NO.14193, 28 × 35 cm, Deli, Zhejiang, China) and sealed using a vacuum machine (PFS-200, Ruixiang, Shanghai, China). Thirty-six bags (4 treatments × 3 ensiling days × 3 replicates) were stored at room temperature (20 ± 2°C). Silage samples were collected at 14, 28, and 56 days of ensiling to evaluate chemical composition, fermentation quality, and microbial community.

**Table 1:** Chemical characterizing and microbial counts of fresh materials.

Items	Corn	Soybean	Corn and soybean (60:40)
DM (%DM)	40.61	30.90	38.49
CP (%DM)	8.64	14.09	12.68
ADF (%DM)	19.43	25.53	21.03
NDF (%DM)	33.4	37.05	36.73
WSC (%DM)	11.05	3.66	5.09
Microorganism (log^10^ cfu/g FM)			
LAB	7.52	4.84	5.12
Yeast	8.01	5.20	5.47
Mold	-	-	-

### 2.2 Aerobic stability test

150 g of silage sample after fermentation for 56 days was transferred to a 200 mL polyethylene (PE) bottle. A multi-channel high-precision automatic temperature recorder (model MDL-1048A, Shanghai Tianhe Automation Instrument Co., Ltd.) with multiple probes was positioned at the feed center to monitor feed and ambient temperature changes every 30 min. Aerobic stability was determined as the duration required for the silage temperature to consistently exceed the ambient temperature by 2°C (Ke et al., 2015).

### 2.3 Fermentation quality, chemical characterizing, and microbial counts of silage

Samples were dried at 65°C for 72 h to measure DM content and subsequently crushed and sieved through a 1 mm mesh for chemical analysis. CP content was determined using the Kjeldahl method, and WSC content was assessed using the anthrone-sulfuric acid colorimetry method (AOAC, 1990). Neutral detergent fiber (NDF) and acid detergent fiber (ADF) contents were analyzed following the method of Van Soest et al. (1991).

A 20 g silage sample was mixed with 180 mL distilled water, homogenized using a high-speed mill at 5000 r/min for 1 min, and then filtered through four gauze layers and qualitative filter paper. The pH was determined using a pH meter (LEICI pHS-3C, Shanghai, China). Lactic acid (LA), acetic acid (AA), and propionic acid (PA) contents were quantified using high-performance liquid chromatography (ICS-3000 system, Dionex, Sunnyvale, CA, USA), as described by Hu et al. (2020). Ammonia nitrogen (NH_3_-N) levels were ascertained following the protocol of Broderick and Kang (1980).

For microbial analysis, a gradient dilution of the filtrate (range 10^−1^ to 10^−6^) was conducted on an ultra-clean bench. A 100 μL sample of the diluted filtrate was plated onto MRS agar for lactic acid bacteria cultivation and incubated at 35–36°C for 3 days before counting the colonies. Additionally, 100 μL of the dilution was plated onto Potato Dextrose Agar plates, incubated at 28°C for 3 days, and then used for counting yeast and mold colonies.

### 2.4 Microbiome sequencing and analysis

To evaluate the effects of additives on fresh material (FM) and silage, 10 g of each was mixed with 40 mL of sterile PBS (pH 7.4). The mixture was then shaken at 120 rpm for 2 h using an electronic oscillator. After filtration through gauze, the filtrate was centrifuged at 13,000 rpm for 10 min at 4°C. The supernatant was discarded, and the pellet was stored on dry ice. Subsequently, an E.Z.N.A.® soil DNA Kit (Omega Biotek, Norcross, GA, U.S.) was used to extract the DNA according to the manufacturer’s instructions. The final DNA concentration and purity were determined using a NanoDrop 2,000 UV–vis spectrophotometer (Thermo Scientific, Wilmington, United States), and DNA quality was assessed by 1% agarose gel electrophoresis.

The V3-V4 region of the 16S rRNA gene was amplified with the primer pair 338F (5′-ACTCCTACGGGAGGCAGCAG-3′) and 806R (5′-GGACTACHVGGGTWTCTAAT-3′). Polymerase chain reaction amplification was conducted as described previously (Hu et al., 2020). Amplified fragments were sequenced on an Illumina MiSeq PE300 platform (Majorbio Biopharm Technology Co., Ltd., Shanghai, China). Raw Fastq files were merged by their overlapping regions with overlaps of >10 bps using Trimmomatic (Bolger et al., 2014). The merged sequences were filtered by removing sequences with a mismatch ratio up to 0.2 and bases containing N to obtain optimized sequences. QIIME (Caporaso et al., 2010) was used to pick operational taxonomic units (OTUs) based on 97% sequence identity and to assess sequence quality. Potential chimeras were removed using Usearch (version 7.1) (Edgar, 2013). OTUs present in negative control amplifications were also removed prior to analysis. Any OTU containing only 1 sequence was removed. The primary OTU sequences were subjected to taxonomic analysis using the Ribosomal Database Project Classifier (version 2.2) and the 16S rRNA database (Silva v138), with a confidence level of 0.7 (Yang et al., 2019). Chloroplast and mitochondrial sequences were removed from further analysis. Alpha diversity and beta diversity were determined by random normalization to the same sequence using QIIME2 (Douglas et al., 2018). Microbial relative abundance was used to represent bacterial classification. We also used linear discriminant analysis effect size (LEfSe) to identify significant associations between bacterial taxa in the treatments (Segata et al., 2011). The 16S rRNA gene sequences of bacterial colonies were annotated and predicted for Kyoto Encyclopedia of Genes and Genomes (KEGG) functions using Tax4Fun (version 0.3.1) (Aßhauer et al., 2015).

### 2.5 Statistical analyses

Statistical analyses were conducted using SPSS 26.0 software (SPSS, Chicago, Illinois, USA) for Windows. Each silage treatment index underwent a one-way analysis of variance (ANOVA), and a general linear model (GLM) was applied for an interaction effect on ensiling days (D) and additive treatment (T). Duncan’s multiple comparison method identified significant differences between treatment means. Effects were considered significant at *p* < 0.05.

## 3 Results

### 3.1 Chemical characterizing and microbial counts of FM

The nutritional content of the soybean-corn whole-plant mix lies between that of soybean and corn, with a DM content of 38.49% FM, WSC content of 5.09% DM, and epiphytic LAB and yeast counts of 5.12 log^10^ cfu/g FM and 5.47 log^10^ cfu/g FM, respectively (Table 1).

### 3.2 Effect of additives on the chemical characterizing in mixed silage

After 14 days of ensiling, the WSC content in CK was significantly higher (*p* < 0.05) than that in LP. After 28 days, the CP content of LP was significantly higher (*p* < 0.05) than that of CK and LPB. After 56 days, the DM and CP contents of LP, LB, and LPB showed an increase compared to CK, but no significant differences were observed among the treatment groups. The WSC content in LB and LP was significantly lower (*p* < 0.05) than that in CK (Table 2).

**Table 2:** Chemical characterizing of whole-plant soybean-corn mixed silage.

Item	Ensiling days	Treatments				SEM (*n* = 3)	*p*-value		
		CK	LP	LB	LPB		T	D	T × D
DM (%FM)	14	37.54	38.7	37.80	38.03	0.574	NS	*	NS
	28	36.31	38.06	37.47	37.40				
	56	36.05	37.36	36.67	36.48				
CP (%DM)	14	11.61	12.25	11.88	11.67	0.208	*	*	NS
	28	11.47b	12.17a	11.80ab	11.64b				
	56	11.26	11.41	11.50	11.39				
ADF (%DM)	14	20.33	20.69	20.42	19.01	1.1019	NS	**	NS
	28	19.60	18.37	18.13	17.75				
	56	19.48	16.38	15.84	17.51				
NDF (%DM)	14	35.05	35.64	36.17	35.97	0.926	*	**	NS
	28	34.66	32.15	33.55	35.64				
	56	33.70	29.88	30.19	32.74				
WSC (%DM)	14	4.62a	2.95b	3.87ab	3.61ab	0.221	**	**	NS
	28	2.43	2.02	2.06	2.05				
	56	2.22a	1.75b	1.83b	1.96ab				

Different letters in the same row indicate significant differences (*p* < 0.05), and the same or no letters indicate non-significant differences (*p* > 0.05). **p < 0.01; *p < 0.05; and NS, *p* ≥ 0.05. CK, no additive; LP, *L. plantarum*; LB, *L. buchneri*; LPB, *L. plantarum* + *L. buchneri*. SEM, standard error of the mean. T, additive treatment; D, ensiling days; T × D, interaction of additive treatment and ensiling days; NS, not significant.

### 3.3 Effect of additives on the fermentation quality in mixed silage

After 14 and 28 days of ensiling, the pH values of the LP, LB, and LPB groups were significantly lower than that of the CK group (*p* < 0.05), and the LB group had a significantly greater AA content than the other groups (*p* < 0.05). After 14 days, the LA content of LP and LPB was significantly higher than that of CK and LB (*p* < 0.05). After 28 days, the NH_3_-N content of LP and LB was significantly lower than that of CK (*p* < 0.05), and LPB had a significantly lower PA content than the other groups (*p* < 0.05). After 56 days of ensiling, the pH value of LP, LB, and LPB remained significantly lower than that of CK (*p* < 0.05). The NH_3_-N content of LB was significantly lower than that of the other groups (*p* < 0.05), and the NH_3_-N content of LP and LPB was significantly lower than that of CK (*p* < 0.05). The AA content of LB was significantly higher than that of CK and LP (*p* < 0.05). However, no significant difference in LA content between the groups was observed (*p* > 0.05) (Table 3).

**Table 3:** Fermentation quality of whole-plant soybean-corn mixed silage.

Item	Ensiling days	Treatments				SEM (*n* = 3)	*p* value		
		CK	LP	LB	LPB		T	D	T × D
pH	14	4.33a	4.07b	4.13b	4.11b	0.027	**	**	NS
	28	4.17a	3.90c	4.02b	3.97bc				
	56	4.03a	3.87b	3.91b	3.93b				
NH_3_-N (%DM)	14	0.17	0.15	0.15	0.16	0.010	**	**	NS
	28	0.24a	0.19bc	0.17c	0.21ab				
	56	0.27a	0.22b	0.18c	0.23b				
LA (%DM)	14	6.68c	9.57a	7.62b	9.31a	0.411	**	**	NS
	28	7.82	9.72	9.18	9.64				
	56	9.66	10.47	9.78	10.15				
AA (%DM)	14	2.99b	2.86b	4.92a	3.29b	0.351	**	*	NS
	28	3.17b	3.13b	5.40a	3.47b				
	56	3.70c	3.66c	6.03a	4.50b				
PA (%DM)	14	0	0.07	0.02	0.04	0.04	*	**	**
	28	0.32a	0.38a	0.25a	0.01b				
	56	0.69	0.66	0.67	0.74				

Different letters in the same row indicate significant differences (*p* < 0.05), and the same or no letters indicate non-significant differences (*p* > 0.05). **p < 0.01; *p < 0.05; and NS, *p* ≥ 0.05. CK, no additive; LP, *L. plantarum*; LB, *L. buchneri*; LPB, *L. plantarum* + *L. buchneri*. SEM, standard error of the mean. T, additive treatment; D, ensiling days; T × D, interaction of additive treatment and ensiling days; NS, not significant.

### 3.4 Effect of additives on the aerobic stability in mixed silage

**Figure 1:**
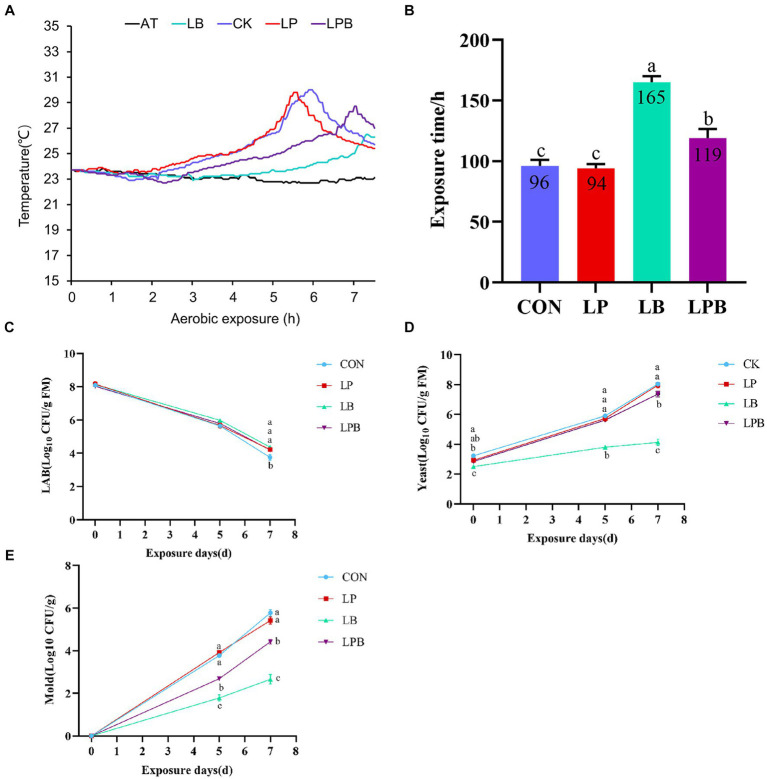
Dynamics of mixed silage temperature (°C) versus ambient temperature during aerobic exposure **(A)** and the time when the silage temperature was 2°C below ambient temperature **(B)**. Dynamics of [**(C)** LAB; **(D)** Yeast; **(E)** Mold] during aerobic exposure. Different letters indicate significant differences (*p* < 0.05), and the same or no letters indicate non-significant differences (*p* > 0.05).

After 56 days of ensiling followed by seven days of aerobic exposure, the aerobic stability time of the silage treated with LB was significantly longer than that of the other groups (*p* < 0.05), as depicted in Figures 1A,B. On the fifth and seventh days of aerobic exposure, the yeast and mold counts in LB-treated silage were significantly lower than those in LP, LPB, and CK (*p* < 0.05), as shown in Figures 1D,E. On the seventh day of aerobic exposure, the counts of LAB were significantly greater in the LP, LB, and LPB treatments than in the CK treatment (*p* < 0.05), as shown in Figure 1C. Furthermore, the counts of yeast and mold in the silage treated with LPB were significantly lower than those in CK and LP (*p* < 0.05), according to Figures 1D,E.

### 3.5 Effect of additives on the microbial diversity in mixed silage

**Figure 2:**
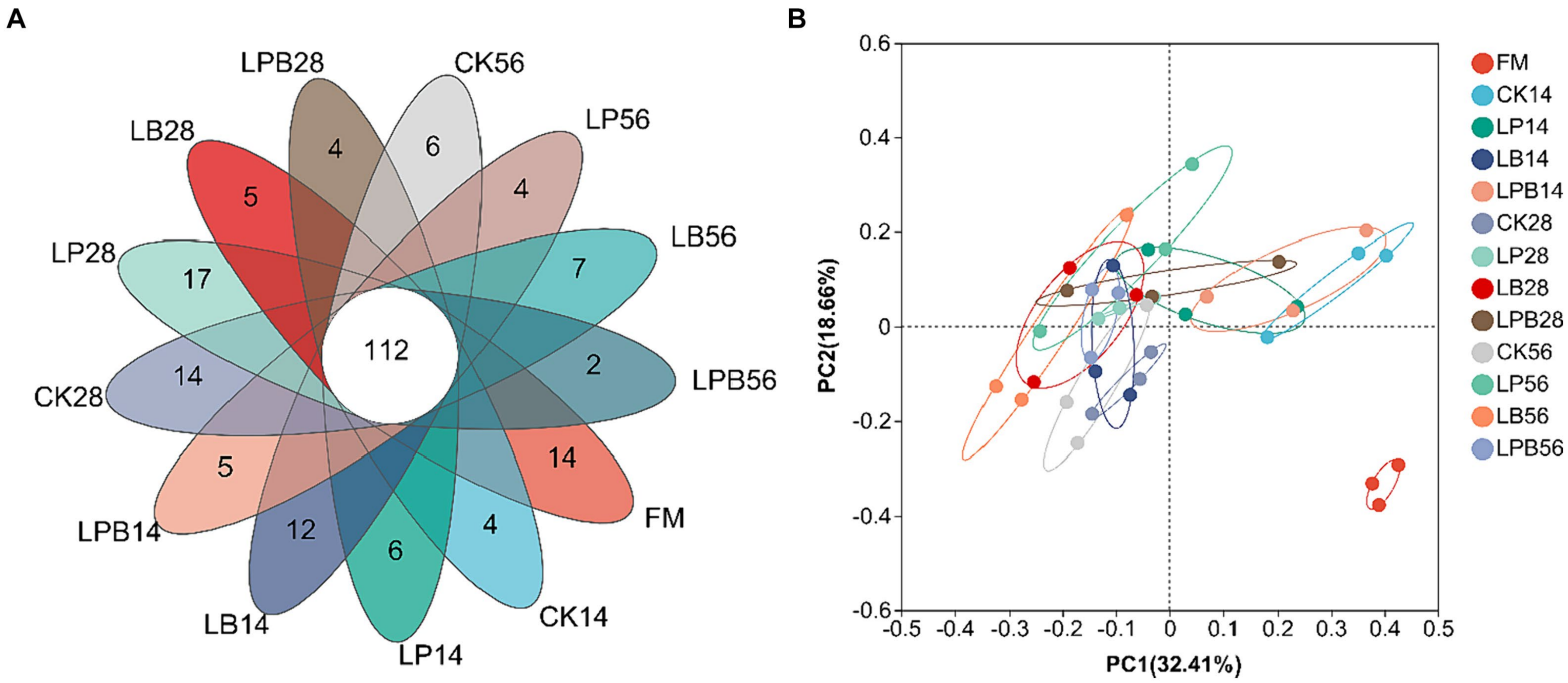
Venn diagram **(A)** and nonmetric multidimensional scaling **(B)** of the bacterial community at the operational taxonomic unit level. FM, fresh material; CK, no additive; LP, *L. plantarum*; LB, *L. buchneri*; LPB, *L. plantarum + L. buchneri*. 14, 28, and 56 indicate 14, 28, and 56 days of ensiling, respectively.

High-throughput 16S rRNA gene sequencing was performed on 39 samples, yielding 1,895,170 valid sequences classified into 659 OTUs. VENN analysis identified 112 shared OTUs across different silage feed groups (Figure 2A). Principal Coordinates Analysis (PCoA) of β-diversity demonstrated clear segregation of bacterial communities between silage and FM (Figure 2B). After 56 days of ensiling, microbial α-diversity did not exhibit significant differences among groups (*p* > 0.05) (Table 4). The coverage index was 0.99, indicating that the sequencing results accurately reflected the microbial community’s characteristics (Table 4).

**Table 4:** Alpha diversity of whole-plant soybean-corn mixed silage.

Items	CK56	LP56	LB56	LPB56	SEM (*n* = 3)	*p* value
Sequence	22,922	19,969	26,997	25,280	6365.09	0.719
OTUS	195	174	177	183	15.94	0.587
Simpson index	0.41	0.31	0.37	0.25	0.06	0.134
Ace index	281	303	300	351	43.46	0.467
Chao 1 index	254	266	259	289	33.10	0.736
Good Coverage index	0.99	0.99	0.99	0.99	0.00	0.369

Different letters in the same row indicate significant differences (*p* < 0.05), and the same or no letters indicate non-significant differences (*p* > 0.05). **p < 0.01; *p < 0.05; and NS, *p* ≥ 0.05. CK, no additive; LP, *L. plantarum*; LB, *L. buchneri*; LPB, *L. plantarum* + *L. buchneri*. SEM, standard error of the mean. T, additive treatment; D, ensiling days; T × D, interaction of additive treatment and ensiling days; NS, not significant.

**Figure 3:**
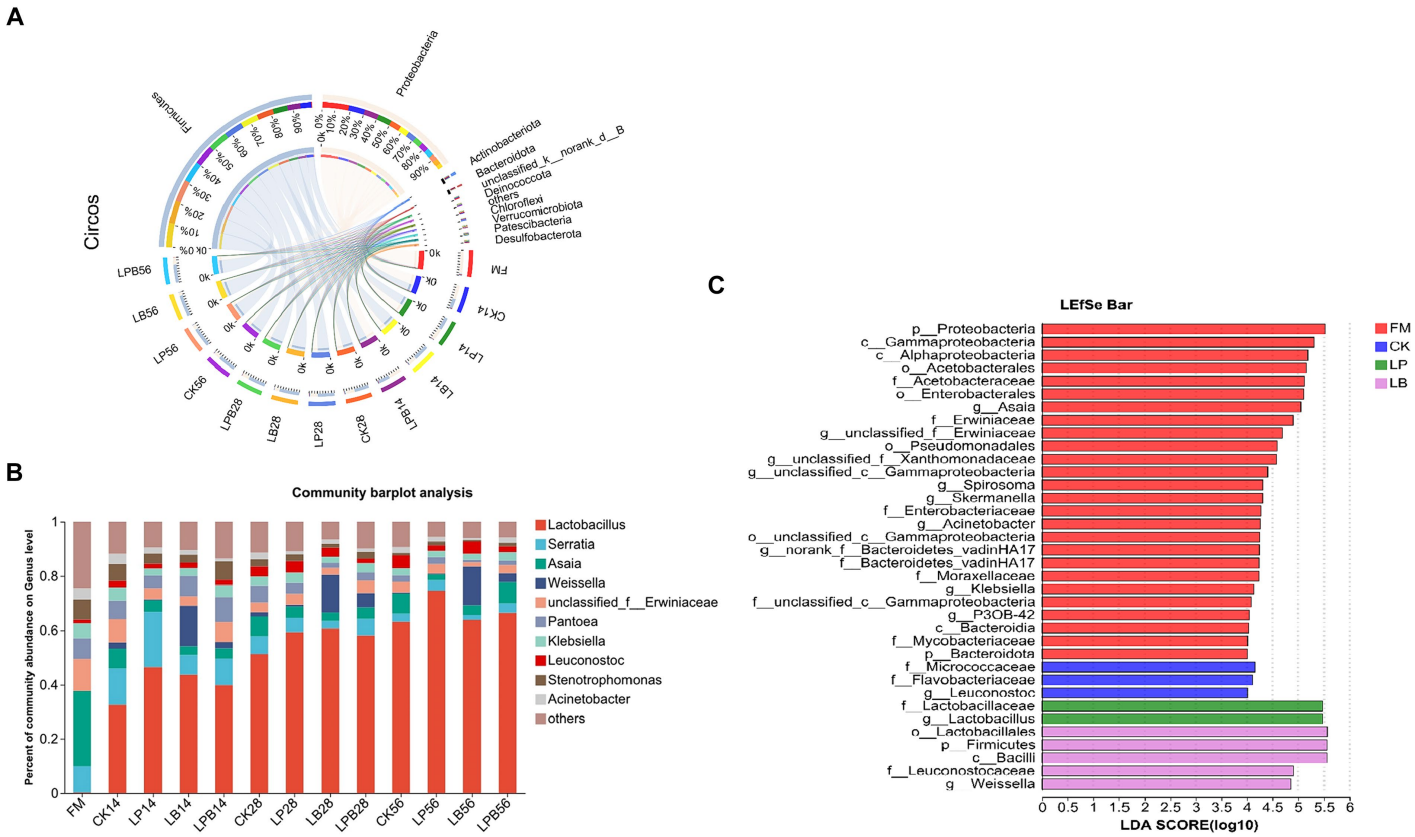
**(A)** The relative abundance (%) of bacterial phyla (at least 1% in one group) in FM and forage oat silage at the phylum level. **(B)** The relative abundance (%) of bacterial phyla (at least 1% in one group) in FM and forage oat silage at the genus level. **(C)** Linear discriminant analysis effect size (LEfSe) was used to assess differences in microbial communities between FM and silage (LDA score > 4.0). The length of the histogram represents the LDA score of different species. FM, fresh material; CK, no additive; LP, *L. plantarum*; LB, *L. buchneri*; LPB, *L. plantarum + L. buchneri*. 14, 28, and 56 indicate 14, 28, and 56 days of ensiling, respectively.

In FM and silage samples, 24 phyla were identified, with the top 10 listed by abundance (Figure 3A). In FM, Proteobacteria dominated (91.78%). After ensiling, Firmicutes became the dominant phylum in all groups, reaching 69.92% in CK56 silage; in LP56, LB56, and LPB56 treated silages, Firmicutes represented 77.79, 84.93, and 72.74%, respectively. A total of 358 genera were detected across the samples, with the top 10 listed by abundance (Figure 3B). The dominant genera in FM included *Serratia* (9.81%), *Asaia* (27.64%), *unclassified_f__Erwiniaceae* (11.72%), and *Pantoea* (7.67%). In silage, *Lactobacillus* predominated. In CK56 silage, *Lactobacillus* (63.12%) and *Asaia* (7.47%) were the main genera; in LP, LB, and LPB treated silages, *Lactobacillus* represented 74.62, 63.82, and 66.42%, respectively, with *Asaia* at 2.11, 3.64, and 7.85%. *Weissella* abundances were 14.88, 13.95, and 14.32% in LB14, LB28, and LB56 treated silages, respectively. LEfSe analysis (LDA = 4) revealed significant taxonomic differences between FM and treatments (Figure 3C), with *g_Asaia* concentrated in the FM group. After ensiling, *g__Leuconostocaceae* was enriched in CK treatment, *g__Weissella* in LB, and *g__Lactobacillus* in LP treatments.

### 3.6 Association between microbial community and fermentation features in mixed silage

**Figure 4:**
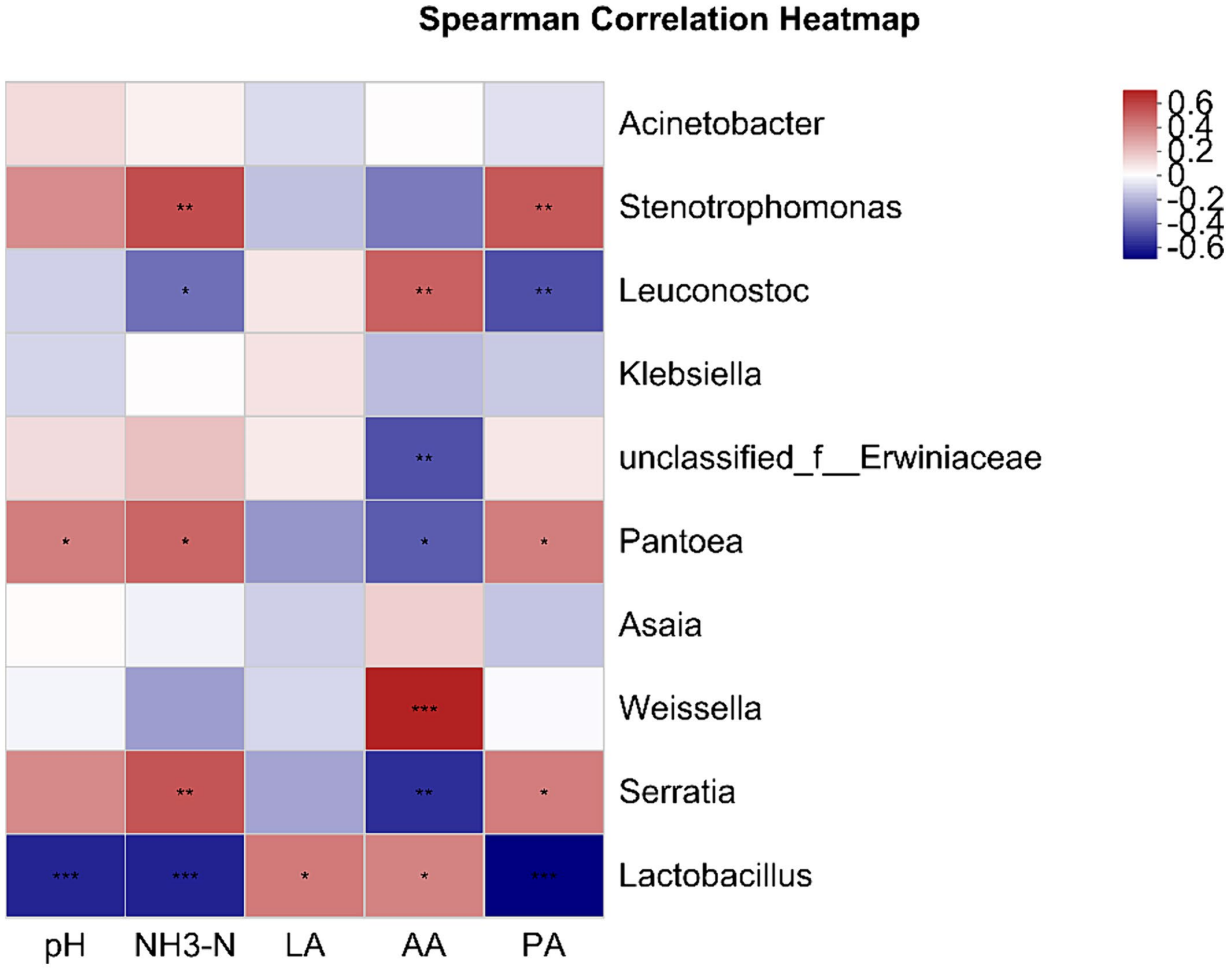
This heatmap displays Pearson’s correlations between genus-level microbial communities and fermentation parameters. Positive and negative correlations are marked in red and blue, respectively. *p < 0.05; **p < 0.01; ***p < 0.001. NH_3_-N, ammonia nitrogen; LA, lactic acid; AA, acetic acid; PA, propionic acid.

Figure 4 illustrates the relationships between silage fermentation parameters and bacterial populations at the genus level. *Lactobacillus* positively correlated with LA and AA, and negatively with pH, NH_3_-N, and PA. AA showed a positive correlation with the genera *Weissella* and *Leuconostoc*. *Serratia*, *Pantoea*, and *Stenotrophomonas* were positively associated with pH, NH_3_-N, and PA.

### 3.7 Predicted functions of the microbial community in mixed silage

**Figure 5:**
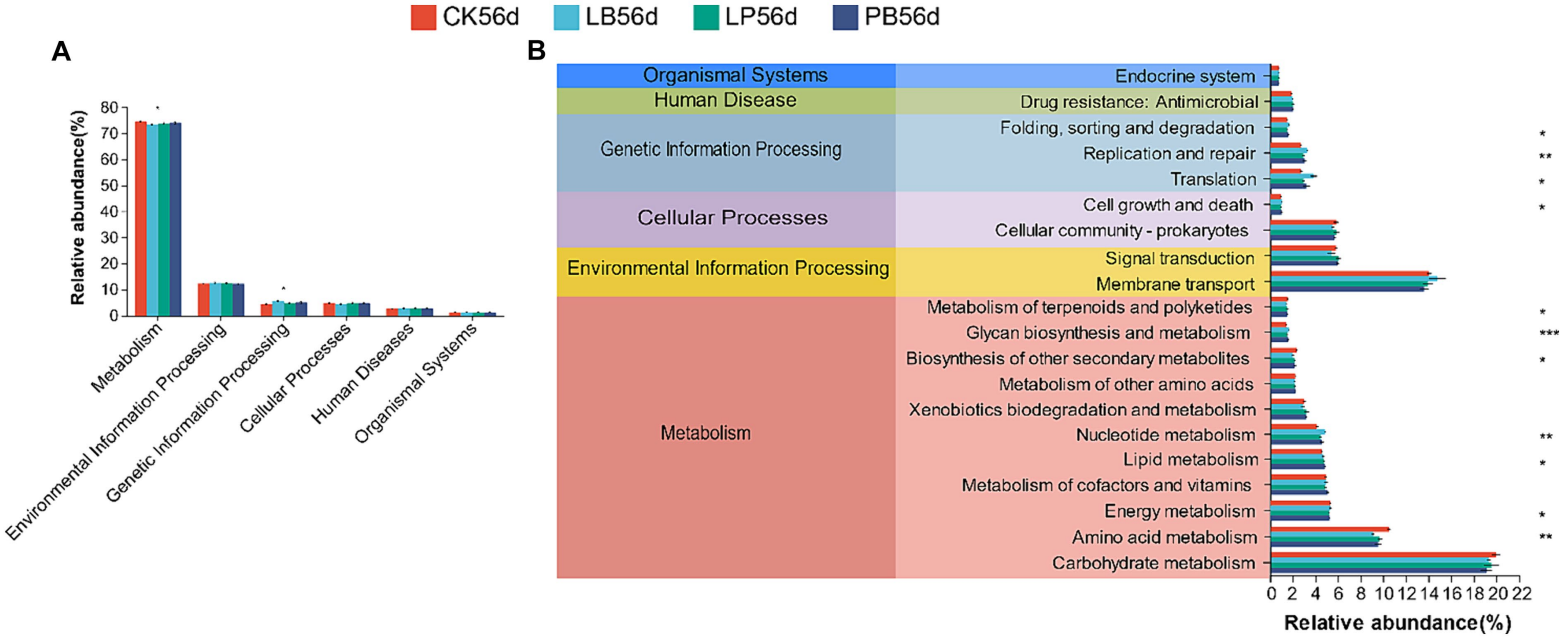
Tax4Fun analysis was used to elucidate the functional metabolic profile of the bacterial community in silage fermented for 56 days. **(A)** Level 1 metabolic pathways; **(B)** Level 2 Kyoto Encyclopedia of Genes and Genomes (KEGG) orthologue functional predictions of the relative abundances of the top 20 metabolic function. *p < 0.05; **p < 0.01; ***p < 0.001. CK, no additive; LP, *L. plantarum*; LB, *L. buchneri*; LPB, *L. plantarum + L. buchneri*. 56 indicates 56 days of ensiling.

Tax4Fun inferred potential functions of the bacterial communities in silage after 56 days of fermentation. Six primary metabolic pathways were identified, with critical predictive genes related to metabolism constituting approximately 73% of the silage genomic repertoire (Figure 5A). The top 20 metabolic pathways in the secondary pathways are listed (Figure 5B). Compared to the control, LP, LB, and LPB showed a significantly lower abundance in amino acid metabolism (*p* < 0.05) and a significantly higher abundance in nucleotide metabolism and sugar biosynthesis and metabolism (*p* < 0.05).

## 4 Discussion

The nutrient contents required for silage production include DM content above 30% (Guyader et al., 2018), WSC content over 5% DM (Ni et al., 2017), and LAB count greater than 5.00 log^10^ CFU/g FM (Cai et al., 1998). After blending whole-plant soybean and corn in a 40:60 ratio, DM, WSC, and LAB counts adjusted to 38.49% FW, 5.02% DM, and 5.19 log^10^ CFU/g FM, respectively, meeting these requirements. The yeast count (5.47 log^10^ CFU/g FM) was higher than LAB, suggesting competition for substrate in early silage stages and indicating the necessity of LAB inoculation in mixed silage.

### 4.1 Effect of LAB on the chemical composition of mixed silage

As anticipated, the DM and WSC content in ensiled material decreased compared to FM and further decreased with silage time. This reduction is primarily due to microorganisms converting nutrients such as DM and WSC into various energy forms during ensiling (Wang S. et al., 2022). After 56 days, the DM contents of LP, LB, and LPB increased by 3.6, 1.7, and 1.2%, respectively, compared to those of the CK group. This shows that vaccination with LAB can inhibit DM loss in WPSCS, which is consistent with the results of Ren et al. (2020) and Xiong et al. (2022). During mixed silage fermentation, LAB and other microorganisms consume WSC, produce organic acids, and reduce WSC content (Fang et al., 2022). Consequently, WSC content is lower in the additive group than in the control group due to significant WSC consumption by LAB during fermentation. In this study, the CP contents of LP, LB, and LPB showed an increase compared to the CK group, correlating with Dong et al. (2022), who found that lower pH inhibits plant protease and microbial activities, retaining CP content.

### 4.2 Effect of LAB on fermentation quality and aerobic stability in mixed silage

Ensiling systems require low pH for feed preservation and inhibiting harmful microorganisms and molds (Zhang et al., 2022). This study aligns with Si et al. (2023), demonstrating an initial rapid pH drop in early silage fermentation, continuing as fermentation progressed. The control silage’s pH (4.03) at 56 days was slightly lower than the pH (4.44) reported by Kizilsimsek et al. (2017) for 60-day corn and soybean silage, likely due to higher WSC content in the whole-plant corn silage. Notably, the pH of silage inoculated with LAB after 56 days of fermentation was significantly lower than that of the control group, highlighting LAB’s effectiveness in silage fermentation. The LP treatment group maintained a consistently low pH during silage, attributed to LP’s role as a homofermentative bacterium that increases LAB population and promotes lactic acid metabolism, thereby accelerating pH reduction (Mu et al., 2020). Proteolysis is a critical issue in silage fermentation, with high NH_3_-N content indicating extensive protein degradation (Tao et al., 2020). This study found that after 56 days of ensiling, the NH_3_-N concentration in the additive-treated silages was lower compared to CK silages, aligning with Dong et al. (2022), who reported LAB’s role in improving silage fermentation quality and reducing protein hydrolysis. The decrease in NH_3_-N content in additive-treated silage may result from the inhibition of Clostridium and other proteolytic bacteria by the low pH environment, thereby preserving the CP content (Dong et al., 2022). Organic acid content and composition patterns serve as essential indicators of silage quality (Jiang et al., 2020). After 14 days of ensiling, the LA content in the LP group surpassed that in the other three groups. However, by day 56, the differences in LA content among all groups were not statistically significant. In the early stages of silage, homofermentative bacteria rapidly consume WSC, leading to high lactic acid production. However, in later stages, limited LAB numbers and WSC content may result in minimal changes in LA (Xu et al., 2019). WSC deficiency induces a shift in fermentation type from homo-to hetero-fermentation, with hetero-fermenters often dominating the ensiling process during extended ensiling periods (Okoye et al., 2023). Compared to AA content (1.3–5.5 g/kg DM) reported in whole-plant corn silage by Huang et al. (2021), this study found that mixed soybean corn whole-plant silage had higher AA content (2.99–6.03 g/kg DM). The addition of whole soybeans may influence the increase in AA content in mixed silage, as studies indicate that low sugar content in legumes induces a shift in LAB fermentation from homo to hetero fermentation (Mu et al., 2020). Hetero-fermentation primarily produces lactic acid and acetic acid, the latter being an effective preservative that inhibits yeast and mold growth. High levels of acetic acid indicate optimal aerobic stability in silage (Arriola et al., 2021). After 56 days of ensiling, silage treated with LB exhibited a higher acetic acid content than the three other groups, significantly higher aerobic stability than the CK and LP groups, and the lowest yeast and mold counts post-exposure. These findings align with those of Keshri et al. (2019) and Wu et al. (2023), confirming that silage supplemented with LB demonstrates superior aerobic stability.

### 4.3 Effect of LAB on the microbial diversity in mixed silage

Studies of various silage microorganisms have shown that Proteobacteria initially dominate in FM but are gradually replaced by Firmicutes post-ensiling (Zhao et al., 2021; Zi et al., 2021; Li et al., 2022), consistent with results of this study. Proteobacteria, the largest bacterial phylum, is succeeded by Firmicutes, which includes a variety of LAB crucial for silage fermentation (Li et al., 2022). After 56 days of ensiling, a higher abundance of Firmicutes was observed in the additive group than in the control, suggesting a higher count of LAB. The majority of epiphytic bacteria in fresh raw materials are not essential for silage, which exhibits a significantly different microbial community composition (Guo et al., 2021). Predominant genera identified in FM in this study were *Serratia*, *Asia*, *unclassified_f_Erwiniaceae*, and *Pantoea*. Post-ensiling, *Lactobacillus* gradually became the dominant genus in each group. *Lactobacillus* is crucial in silage, producing LA, reducing pH, suppressing undesirable bacteria, and often dominating in high-quality silage (He et al., 2019; Li et al., 2021). The relative abundance of *Lactobacillus* was higher in the inoculated group than in the control, likely due to LAB addition fostering a conducive environment for LAB growth. This is supported by findings of Ni et al. (2017) and Wang Q. et al. (2022), who reported increased *Lactobacillus* abundance and altered microbial community structure in silage following LAB inoculation. *Weissella*, a hetero-fermentative LAB, colonizes early in silage. As pH decreases, it is progressively replaced by acid-resistant lactobacilli (Wang et al., 2019). LB-treated silages in this study consistently showed high *Weissella* abundance, potentially due to LB’s enhancement of *Weissella* competitiveness during silaging. Bai et al. (2020) observed a similar increase in *Weissella* abundance in LB-treated alfalfa silage after 60 days of fermentation. Additionally, LEfSe results indicated that *Leuconostoc* was enriched in CK groups, while *Weissella* was enriched in the LB group. Both primarily perform hetero-fermentation, converting various organic compounds into acetic acid (Chun et al., 2017), contributing to elevated acetic acid levels in CK and LB silage groups.

During the anaerobic fermentation process, microorganisms such as LAB play an indispensable role. They convert nutrients into volatile fatty acids and play a key role in improving silage quality and aerobic stability (Holzer et al., 2001). In this study, a positive correlation between *Lactobacillus* and LA and AA was shown by Spearman correlation analysis. This finding is consistent with the results of Sun et al. (2021) on whole plant corn fermentation patterns, further confirming the importance of lactic acid bacteria in the production of LA and AA. Similarly, the production of acetic acid is closely related to the activities of *Weissella* and *Leuconostoc*, which serve as hetero-lactic acid bacteria in silage fermentation and are mainly responsible for the production of LA and AA (Wang et al., 2019). The increased AA content in the LB group silages and the high abundance of these two bacterial genera may be the reasons for the prolonged aerobic stability of the LB group. Enterobacter can not only convert LA to AA and other organic acids but also metabolize protein to NH3-N, resulting in poor silage fermentation (Spoelstra, 1987). The positive correlation between the abundances of *Serratia* and *Pantoea*, which are members of the Enterobacteriaceae family, and NH_3_-N in this study further confirmed the protein-degrading role of Enterobacteriaceae. Some studies have shown that microbial metabolism results in nutrient consumption during silage. Fermentation of Enterobacteriaceae leads to the degradation of protein; it can ferment LA or glucose to AA and BA under anaerobic conditions, and Enterobacter and Lactobacillus also compete for limited nutrients; therefore, Enterobacter is not conducive to maintaining the fermentation quality and nutrients of mixed silage (Zong et al., 2023).

Despite high-throughput sequencing technology’s ability to analyze microbial community structure and diversity, it does not directly predict changes in bacterial community functions. To address this, Tax4fun, a tool for predictive microbial community functions, was utilized in this study to predict the KEGG metabolic profiles of bacterial communities within silage samples. The most abundant level 1 pathway was metabolic, aligning with previous findings that bacteria in silage efficiently convert substrates into various metabolites, leading to richer metabolic pathways (Xiao et al., 2022). Amino acids, vital for protein synthesis and primary metabolism in soybean-corn mixed crop silage, should be preserved from excessive catabolism during fermentation (Wang Y.-L. et al., 2022). At pathway level 2, the abundance of the amino acid metabolic pathways of LP, LB, and LPB was lower than that of the control group, which further explains the lower ammonia nitrogen levels in the inoculated groups. This suggests that LAB addition can inhibit mixed silage protein hydrolysis, likely due to lower pH, as demonstrated by Flythe and Russell (2004), who noted that acidic fermentation conditions can interfere with amino acid metabolism in forage. Nucleotides, crucial for DNA synthesis, energy provision, and participation in biochemical and regulatory reactions in bacteria (Bai et al., 2022), showed enhanced metabolism in the inoculated group compared to the control, as evidenced by Zhao et al. (2023). This enhancement in nucleotide metabolism in the inoculated group may be attributed to increased LAB relative abundance during silage processing. Furthermore, sugar biosynthesis and metabolism were significantly higher in the inoculated group, correlating with findings by Hisham et al. (2022). However, linking sugar biosynthesis to metabolism and fermentation requires further verification through histological studies such as transcriptomics, proteomics, and metabolomics.

## 5 Conclusion

After inoculation with lactic acid bacteria, the fermentation quality of whole-plant soybean and whole-plant corn mixed silages was satisfactory. This study showed that inoculation with LAB increased the lactic acid content of mixed silage while decreasing the pH, dry matter loss, and NH_3_-N content. Moreover, the aerobic stability time of mixed silages inoculated with LB was prolonged. *Lactobacillus* was relatively abundant in the LP-treated silages, and *Weissella* was relatively abundant in the LB-treated silages. Gene function prediction results showed that the metabolic abundance of amino acids in the mixed silage inoculated with LAB was lower than that in the CK group, indicating that adding LAB may inhibit the degradation of proteins in the mixed silage.

## Data availability statement

The datasets presented in this study can be found in online repositories. The names of the repository/repositories and accession number (s) can be found at: https://www.ncbi.nlm.nih.gov/sra/PRJNA1046156.

## Author contributions

JX: Investigation, Writing – original draft, Writing – review & editing. JM: Data curation, Writing – review & editing. RS: Data curation, Writing – review & editing. HS: Data curation, Writing – review & editing. XW: Data curation, Writing – review & editing. QL: Data curation, Writing – review & editing. XZ: Data curation, Writing – review & editing. BW: Data curation, Writing – review & editing. ZH: Data curation, Supervision, Writing – review & editing. HN: Funding acquisition, Project administration, Supervision, Writing – review & editing.
